# Intrapopulation adaptive variance supports thermal tolerance in a reef-building coral

**DOI:** 10.1038/s42003-022-03428-3

**Published:** 2022-05-19

**Authors:** Crawford Drury, Nina K. Bean, Casey I. Harris, Joshua R. Hancock, Joel Huckeba, Christian Martin H, Ty N. F. Roach, Robert A. Quinn, Ruth D. Gates

**Affiliations:** 1 Hawaiʻi Institute of Marine Biology, University of Hawaiʻi, Kāneʻohe, HI USA; 2 University of Amsterdam, Amsterdam, Netherlands; 3 Department of Biochemistry and Molecular Biology, Michigan State University, East Lansing, MI USA

**Keywords:** Ecological genetics, Animal breeding

## Abstract

Coral holobionts are multi-species assemblages, which adds significant complexity to genotype-phenotype connections underlying ecologically important traits like coral bleaching. Small scale heterogeneity in bleaching is ubiquitous in the absence of strong environmental gradients, which provides adaptive variance needed for the long-term persistence of coral reefs. We used RAD-seq, qPCR and LC-MS/MS metabolomics to characterize host genomic variation, symbiont community and biochemical correlates in two bleaching phenotypes of the vertically transmitting coral *Montipora capitata*. Phenotype was driven by symbiosis state and host genetic variance. We documented 5 gene ontologies that were significantly associated with both the binary bleaching phenotype and symbiont composition, representing functions that confer a phenotype via host-symbiont interactions. We bred these corals and show that symbiont communities were broadly conserved in bulk-crosses, resulting in significantly higher survivorship under temperature stress in juveniles, but not larvae, from tolerant parents. Using a select and re-sequence approach, we document numerous gene ontologies selected by heat stress, some of which (cell signaling, antioxidant activity, pH regulation) have unique selection dynamics in larvae from thermally tolerant parents. These data show that vertically transmitting corals may have an adaptive advantage under climate change if host and symbiont variance interact to influence bleaching phenotype.

## Abstract (web summary)

Selective breeding of corals with different bleaching phenotypes demonstrates the potential for climate adaptation in vertically transmitting species.

## Introduction

Reef-building corals are complex holobionts composed of a cnidarian host, symbiotic dinoflagellates in the family *Symbiodiniaceae* and a suite of other micro-organisms. Each compartment of the holobiont interacts to dictate trait values like thermal tolerance, which is increasingly important for ecosystems facing climate change. Corals are susceptible to heat stress, which leads to the breakdown of the coral-algal symbiosis and compromises the structure and function of reefs^1,2^. Despite these threats, there is substantial variation in thermal tolerance within and among populations that can exist over very small spatial scales^3–9^ and supports the adaptive potential of reef-building corals^10–13^. This variation is driven in part by symbiosis with diverse dinoflagellates in the family Symbiodinacea^14–16^, which have strong effects on holobiont physiology. The microbial consortia and its interactions with symbionts^17^ and the host also impact holobiont performance^18,19^ and may significantly complicate genotype-phenotype connections in corals.

Host genomic variation is an important driver of coral bleaching^3,20,21^, but previous research on the adaptive capacity of the coral host has focused almost exclusively on differences driven by strong selection across obvious environmental gradients^5,20–24^. This framework has also been leveraged in breeding experiments with mostly aposymbiotic larvae^12,13,21^, limiting our understanding of the adaptive potential of reefs over smaller scales (e.g., single reefs) where fertilization is most likely to occur. Within these more subtle environmental contours, variation in coral bleaching produced by symbionts and other demographic processes is still ubiquitous^25^.

Stable, heritable variation in thermal tolerance may be created by genomic variants which directly impact performance or indirectly influence symbiont community in brooding or vertically transmitting corals^26^, representing an alternate genotype-phenotype connection established via the symbiosis. These factors represent a mostly unknown reservoir of resilience in the absence of a priori environmental correlates that may be increasingly important as reefs become more isolated in climate refugia^27^.

*Montipora capitata* is a cosmopolitan reef-building coral that is dominant in Kāneʻohe Bay, Oʻahu, Hawaiʻi. *M. capitata* is a vertically transmitting broadcast spawner which releases symbiont provisioned eggs, creating a tight link between host and symbiont^26,28^ which may facilitate ‘adaptive’ change by additive, diversified sources of resilience. Maternal inheritance of symbionts could also serve as a selective pressure on paternal genes that increase fitness when paired with specific types of *Symbiodiniaceae*, fortifying the intergenerational connection between host and algae within individual holobionts. Bleaching phenotype in this *M. capitata* population is primarily driven by symbiosis with *Cladocopium* or *Durusdinium*^29,30^, where individual corals typically harbor *Cladocopium* and are more thermally sensitive (*‘*bleached’ phenotype) or a mixed community of both genera and are more thermally tolerant (‘nonbleached’ phenotype)^31^. Symbiont communities in this population also covary with host pigments^32^ and microbial communities^33^ and are broadly defined by depth and other environmental cues^31^. There are infrequent exceptions to these patterns^29^, but these relationships appear broadly fixed through time and multiple bleaching events^30^, suggesting that a functional connection between host genotype and symbiont constituency minimizes symbiont ‘shuffling’. Nested within this symbiont-phenotype framework, there is a strong host genotype effect on the biochemical signature of metabolites in these corals, including those generated by the symbionts^34^, highlighting the deep connection between the host and symbiont biochemistry *in hospite*.

This framework allows the ultimate phenotype in our study, bleaching tolerance, to have multivariate drivers including cnidarian heat-stress response, symbiosis maintenance physiology, other microbial effects and their interactions. We evaluated transmission of symbiont communities, intrapopulation genomic variance and the consequent biochemical signatures that define coral thermal tolerance. We then bulk crossed gametes from each parental coral phenotype, documenting improved thermal tolerance in juveniles from tolerant parents, and used a select and re-sequence approach to define genomic variants associated with improved thermal tolerance in larvae, directly or via their impact on the symbiosis.

## Results

### Adult population genetics and symbioses

**Fig. 1:**
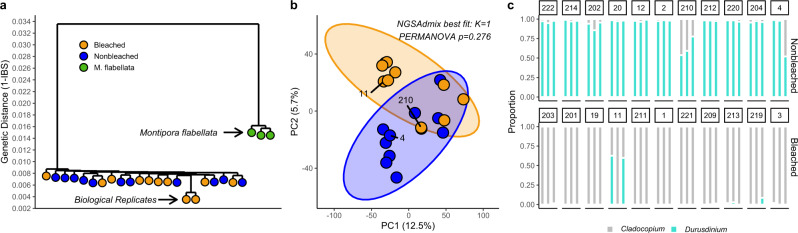
Genomic and symbiotic dynamics underly bleaching phenotype in Montipora capitata. **a** Hierarchical clustering of genetic distances calculated from genotype likelihoods, with one sequenced pair of biological replicates to confirm the absence of clonality and 3 M. flabellata. **b** PCA of 20,407 loci, shaded by historical bleaching phenotype. **c** Symbiont communities in three haphazard replicates of each colony used in this study, shaded by proportion of Cladocopium or Durusdinium measured via qPCR.

Adult coral phenotype in this system was driven by symbiont community and association with host genomic loci. Adult corals in this study were unique genotypes (Fig. 1a) from a single population (F_ST_ = −0.0029; *NGSadmix* K = 1). Phenotype explained 5% of variation, but was not a significant factor defining overall genetic patterns (PERMANOVA *p* = 0.276; Fig. 1b). Genetic distances between samples of *M. capitata* were approximately 4x smaller than between samples of *M. capitata* and *M. flabellata* from Kāneʻohe Bay. Admixture analysis resolved a single population and an analysis of 2 populations showed no signature of coral phenotype in admixture proportions.

Corals from the nonbleached phenotype were dominated by *Durusdinium*, frequently with background levels of *Cladocopium* at <10% abundance (Fig. 1c). Conversely, all Bleached phenotype corals harbored primarily *Cladocopium*, except colony 11 which contained *Durusdinium* in 2 replicates. Replicate sampling suggested occasional heterogeneity in symbiont genera within colonies. There were several exceptions to this pattern: colony 210 and 4 harbored substantial *Cladocopium* communites while colony 11 contained nearly 50% *Durusdinium* (Fig. 1c). We took advantage of these exceptions to parse binary phenotype effects from host-symbiont effects which subsequently have a strong role in phenotype using association testing.

### Genetic association with phentoype and symbiont community

We examined genetic association of individual loci with binary phenotype assignment (Bleached, Nonbleached) and detected no private alleles and no fixed loci, similar to^21^, suggesting incomplete segregation of the phenotypes. We detected 36 loci (0.176% of loci analyzed) significantly associated with phenotype (*p* < 0.0001; Supplementary Table 3). We used the LRT values for 20,407 loci as ‘heats’ to find functional groups corresponding to binary phenotype. Several ontologies (*n* = 28) were significantly enriched in loci with high association values (FDR *p* < 0.1), indicating an elevated influence on phenotype compared to background. These ontologies included protein metabolism, localization and modification, GTPase activity, ribosomal structure, cell-signaling/Erk Cascades, exopeptidase activity and endosomal membranes (Supplementary Table 4). Conversely, steroid metabolism, innate immune response, developmental regulation, cell cycling, downregulation of cell growth, the endoplasmic reticulum, ds-DNA binding, coreceptor binding and calcium ion binding were enriched in low LRT values, suggesting a significant similarity between phenotypes in certain functions or processes that do not influence thermal tolerance (*n* = 48 ontologies; Supplementary Table 5).

**Fig. 2:**
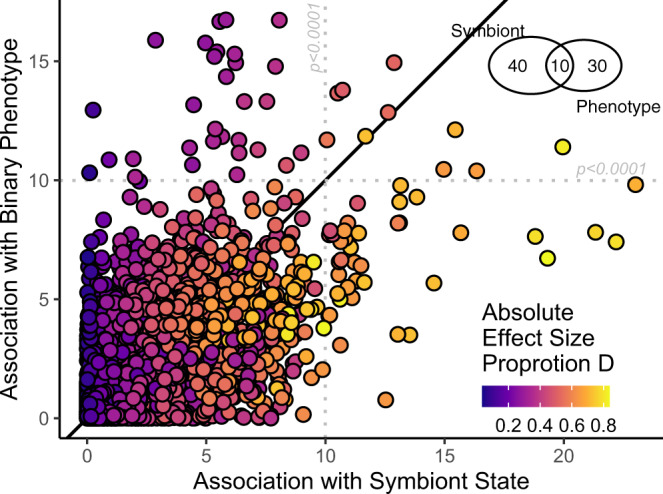
Comparison of genetic associations with binary phenotype and symbiont state. Points represent LRT values from two association tests: case-control association by allele frequencies using phenotypes as the dependent variable and continuous association by gene dosage using proportion Durusdinium as the dependent variable. Points (*n* = 10,803) represent loci shared between the two analyses, excluding those which were filtered to complete continuous association. Point color represents absolute effect size (beta) from symbiont association. Gray lines represent independent LRT value thresholds at *p* = 0.0001 determined by 10 k permutations. Venn diagram contains counts of these significant loci for phenotype, symbionts and shared between the two. Black line is *x* = *y*.

**Fig. 3:**
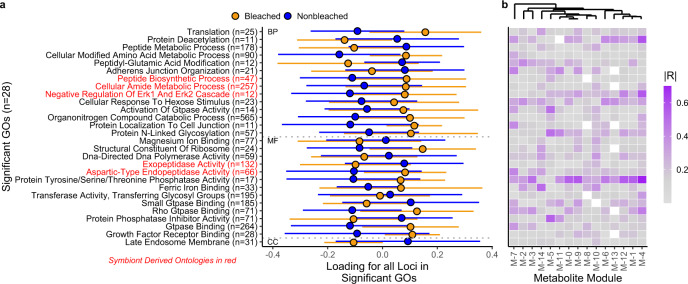
Functional ontologies distinguish bleaching phenotype and relate to biochemical patterns. **a** The 28 gene ontologies that were significantly enriched in loci highly associated with bleaching phenotype (FDR *p* < 0.0001). Points represent each phenotype in the study (mean ± 1 SE) along the first principal component that describes all loci associated with the ontology of interest. Number of loci corresponding to each ontology are in parentheses, red ontologies are significantly enriched in loci with high associations with symbiont state and thus impact phenotype via symbiosis. **b** WGCNA modules of the corals in this study, shaded by correlation with loading in (**a**). Each column represents a metabolite module resolved by WGCNA and each row corresponds to gene ontologies to the left. No correlations were significant after FDR correction.

We then examined genetic association with mean proportion *Durusdinium* as the dependent variable, using a subset of loci which passed continuous association filtering requirements (*n* = 10,803). This analysis leverages exceptions to phenotype-symbiont patterns to decouple a priori phenotype assignments from association testing. Although binary phenotype and symbiont association LRT values were correlated (lm *p* < 0.001, *R*^2^ = 0.408), 75% of the loci important for distinguishing binary phenotype were not significantly associated with symbiont state (Fig. 2 inset). We repeated the GO_MWU enrichment analyses using effect sizes and LRT as heats, concatenated outputs and found 40 gene ontologies significantly enriched in loci with which determined symbiont state (Supplementary Table 6). Of the 28 ontologies associated with overall phenotype, only 5 (17%) were also significantly enriched in the symbiont association testing analysis: Peptide Biosynthetic Process, Cellular Amide Metabolic Process, Regulation of Erk1 and Erk2 Cascade, Exopeptidase Activity and Aspartic-Type Exopeptidase Activity (Fig. 3a red GOs).

For gene ontologies significantly enriched in high association values for phenotype, we extracted all loci annotated as each GO (*n* = 11–565; Fig. 3a) and used *PCAngsd* to summarize genetic variation in these functions. This genetic variation broadly impacted the biochemistry of corals in this study (Fig. 3b), especially in functions related to Protein Deacetylation, Erk Cascade Regulation and Protein Tyrosine/Serine/Threonine Phosphatase. Erk Cascade Regulation was also an ontology that significantly described symbiont state.

### Genetic-metabolomic correlates

There were high correlations between metabolite Module-4 and each of these gene ontologies (R > 0.5, *p* < 0.0005). While these correlations were not significant after multiple comparisons correction, the conserved response of M-4 with multiple phenotype-defining gene ontologies suggests that this pattern is biologically important. Module-4 was significantly enriched in peptides, dipeptides, N-acyl-alpha amino acids and Trifluoromethylbenzenes (FDR *p* < 0.03). Module-5 was also highly correlated (R > 0.5) with Protein Phosphatase Inhibition Activity, Protein N-linked Glycosylation and GTPase activity and significantly enriched in menaquinones and prenol lipids (FDR *p* < 0.02). The betaine lipid identified in^34^, an important molecule for distinguishing phenotype in this species, was in Module-7.

### Betaine glycine

Among the top 36 individual loci for defining phenotype (LRT permutation p < 0.0001), two were on Chromosome 11 separated by approximately 11 kb (Chr11_4203672 and Chr11_4215008). These loci were strongly linked (GL R^2^ = 0.87), suggesting coinheritance and selection on nearby genes. We annotated a 50 kb window centered on the variants of interest (following^21^) using blastn. Several predicted genes were within this window, including glycine sarcosine dimethlycine N-methyltransferase-like (GSMT/SDMT, e = 2e-^105^). The product of GSMT/SDMT is Betaine Glycine (BG), which we documented in our metabolite dataset. BG was more abundant in nonbleached corals than their bleached counterparts (Wilcox test *p* = 0.005; Supplementary Fig. 4); however, colony 203 was an extreme outlier in the bleached phenotype. If this colony was excluded, concentrations were 22.8% higher in Nonbleached corals (Wilcox test *p* < 0.001). We examined loci annotated as ‘Amino-acid Betaine Metabolic Process’ (GO: 0006577, *n* = 10) with a Wilcox test and found a significantly higher LRT than other ontologies (*p* = 0.013) in the binary phenotype analysis, suggesting this ontology is enriched in loci that differentiate phenotypes and symbiont communities. The effect size derived from association testing of symbiont state was 26.8% larger in this window than across the rest of the genome (Wilcox *p* < 0.001). These loci were not included in the association testing for symbiont type due to data quality filtering.

### Larval and juvenile survivorship

**Fig. 4:**
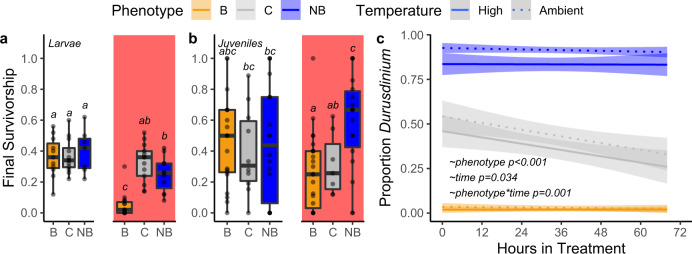
Fitness and Symbiont Dynamics in Larval and Juvenile corals. **a** Final survivorship 213 h post fertilization for larvae at ambient and high temperatures. **b** Final survivorship 34 days post settlement for juveniles at ambient and high temperatures. Boxplots are mean 1± IQR, red fill denotes high temperature treatments. **c** Proportion Durusdinium of the three phenotype larval pools from fertilization to 84hpf in ambient and high temperatures. Line is best-fit with 95% confidence interval. Letters represent post hoc significance values for each life-history stage.

To assess the adaptive potential of this symbiotic and genetic variation, we selectively crossed gametes from the adult corals on 13 July 2018, when all 22 colonies in the study spawned. A total of 11 colonies of each phenotype were bulk fertilized, allowing for up to 110 potential crosses in each pure phenotype and 462 crosses in the site-wide control, excluding selfing. Larval survivorship was significantly higher at ambient temperatures throughout the timeseries (Supplementary Fig. 5; Supplementary Table 7; P_LRT_ < 0.01) and at the endpoint (Fig. 4a; *p* < 0.001). There were no significant differences in endpoint survivorship at ambient temperatures between phenotypes (*p* > 0.99). Final survivorship averaged between 36.4–40.0% in the ambient treatments.

Larvae produced from bleached parents had substantially and significantly lower survivorship (4.9 ± 2.0%; mean ± 1SE) than larvae from nonbleached parents (24.9 ± 3.4%, ~5-fold difference, *p* < 0.001) and the cross phenotype (32.6% ± 3.4%, *p* < 0.001). At high temperatures, survivorship in the cross treatment was ~7% higher than the nonbleached phenotype but was not significantly different (*p* = 0.697). Phenotype explained 96.3% of survivorship variance in high treatments (H^2^ = 0.963), and 17.3% in ambient treatments (H^2^ = 0.173).

In 1.5-month-old juveniles, survivorship was significantly higher at ambient than high temperatures throughout the timeseries (Supplementary Fig. 6; Supplementary Table 8; P_LRT_ < 0.01) and at the endpoint (Fig. 4b; *p* = 0.040). There were no significant differences in ambient endpoint survivorship between phenotypes (*p* > 0.99). Nonbleached juveniles had higher survivorship than the site-wide cross (*p* = 0.048) or bleached phenotype (*p* = 0.001, ~2-fold difference) under heat stress (Fig. 4b). Final survivorship under heat stress for the cross and bleached phenotype were not significantly different (*p* = 0.721). Further, nonbleached juvenile survivorship under temperature stress was not significantly different than either pure phenotype in ambient condition (*p* > 0.19). Phenotype explained 92.9% of variance in high treatments (H^2^ = 0.929), and 0.2% in ambient treatments (H^2^ = 0.002).

*M. capitata* releases symbiont provisioned eggs which contribute strongly to downstream thermal tolerance, so we also quantified symbiont community over time in larvae using qPCR. Treatment did not have a significant main effect on symbiont community (beta-regression; $\chi$^2^ = 1.62, *p* = 0.203). The symbiont communities were strongly differentiated between phentoypes (beta-regression; $\chi$^2^ = 146.6, *p* < 0.001) and there was a significant interaction of phenotype, treatment and time (beta-regression; $\chi$^2^ = 13.2, *p* = 0.040) with *Durusdinium* or *Cladocopium* dominating the nonbleached and bleached larvae, respectively (Fig. 4c). Nonbleached larvae typically had background levels of *Cladocopium*, but bleached larvae typically did not have any *Durusdinium*. The site-wide control cross has an approximately equal proportion of each genus at the initiation of heat stress, but a substantial decline in proportion *Durusdinium* occurred in the ambient treatments of the control cross (beta-regression, z-ratio = 3.60, *p* = 0.056); while this pattern was very similar in the high treatment, the only significant declines occurred between 0 and 44 h (beta regression, z-ratio = 6.04, *p* < 0.001). The bleached (beta-regression, z-ratio < 2.28, *p* > 0.82) and nonbleached (beta-regression, z-ratio < 1.41, *p* > 0.99) crosses remain dominated by their initial symbiont communities.

### Larval selection patterns

We used a select and re-sequence approach in larvae in this study to define genetic response to heat selection, examining the PBS of 31,794 loci shared between the site-wide cross and nonbleached pools. PBS values from the control phenotype explained 33.2% of variance from the nonbleached phenotype (lm *p* < 0.001, Supplementary Fig. 7), but mean PBS was 65% higher in the cross phenotype compared to nonbleached (Wilcox *p* < 0.001).

**Fig. 5:**
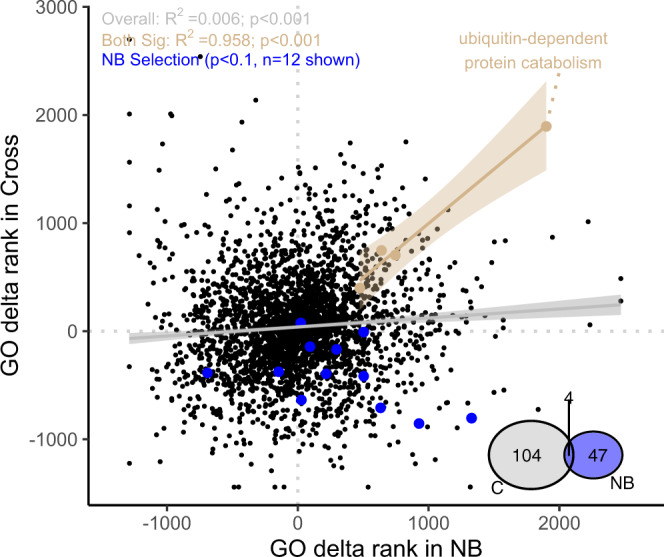
Selection Dynamics in Larval heat stress experiment. GO delta rank derived from PBS statistics of cross and nonbleached larval pools. High values represent gene ontologies enriched in large PBS statistics. Tan points represent the 4 gene ontologies that were significant (FDR *p* < 0.1) in both cross and nonbleached phenotypes, with lm best fit line. Black points represent the ontologies that were not significant in both treatments, with gray lm best fit line. Blue points represent gene ontologies with enrichment in Nonbleached phenotype and delta rank <100 (not significant) In the cross phenotype, isolating the genomic effect of selective breeding. Venn diagram shows shared significant ontologies between phenotypes.

After mapping these loci and retrieving gene ontologies, there were 108 and 51 ontologies significantly enriched for selected loci (high PBS values) in the site-wide cross and nonbleached phenotypes, respectively. Four of these significant functions were shared between phenotypes (FDR *p* < 0.1, Fig. 5 inset): oxidation-reduction processes (GO:0055114), stress-activated protein kinase signaling cascades (GO:0031098), positive regulation of cell-cell adhesion (GO:0022409) and ubiquitin-dependent protein catabolic processes (GO:0043162). Overall, GO ranks within each phenotype were significantly correlated in the global dataset, but had virtually no explanatory power (Fig. 5 gray/black; lm *p* < 0.001, R^2^ = 0.006). The four ontologies that were significantly enriched in selected for both phenotypes were highly coordinated (Fig. 5 tan; *n* = 4, lm *p* < 0.001, R^2^ = 0.985).

Twenty functional categories were significantly enriched for large difference between PBS values in the nonbleached and cross phenotypes, representing heat selection specific to the thermally tolerant population. These categories included antioxidant activity, protein serine/threonine/tyrosine kinase activity, nonsense mediated decay regulation and several ontologies related to pH regulation (Fig. 5 blue points, Supplementary Table 9).

## Discussion

The long-term persistence of coral reefs requires adaptive differences based on heritable variation which keeps pace with a changing climate. Here we demonstrate a shared host genomic and symbiotic foundation for intrapopulation variance in thermal tolerance on a single reef in the absence of differential selection. These additive sources of resilience lead to increased thermal tolerance in the juvenile stage of subsequent generations and produce high heritability values which may contribute to adaptation on reefs. Although only ~20% of broadcast spawning corals are vertical transmitters^35^, these species may have an adaptive advantage under climate change if host and symbiont components of the holobiont interact to contribute to phenotype.

*M. capitata* colonies in Kāneʻohe Bay exist over a genetic and environmental mosaic^36^ encompassing a range of symbiont states^31^ and microhabitats. To focus on bleaching phenotype, we identified colonies from the extremes of the thermal tolerance continuum, representing non-random selection of corals within the population. Despite skewing our collections in this manner and the strong symbiotic and biochemical differentiation between phenotypes^30,34^, we found low population stratification and relatively little genome-wide variance explained by phenotype. To eliminate the potential for cryptic speciation, which can align with thermal tolerance and symbiont communities (including in Montiporids)^37–39^ we compared our samples to the congener *M. flabellata* from Kāneʻohe Bay. Each population structure approach used here, including admixture analysis, FST calculations, PERMANOVA and interspecific distances indicated corals were from a single population of *M. capitata*, with genetic differentiation at least four-fold smaller than the distance between species.

The low genetic differentiation between phenotypes simplifies association testing within species, which we conducted for two traits: binary phenotype assigned during the 2015 bleaching event and proportion *Durusdinum*. Many canonical heat-stress response pathways distinguished these phenotypes and symbiont communities. This result in the absence of differential selection (e.g., across environmental gradients) supports the adaptive capacity of phenotypic variation over very small scales, directly or proximally via symbiont community. Ontologies relating to growth/development (GO:0005138;GO:0070851), cell-signaling (GO:0008138), ribosomal constituents (GO:0003735), GTPase activity (GO:0051020) and protein localization, modification and breakdown (GO:0006518; GO:0043043; GO:0043603) have been documented extensively in previous work on both genomic and transcriptomic impacts of thermal tolerance^25,40^. We documented a single cellular compartment, the late endosome which is part of the response to a broad variety of environmental stressors in corals^40^. Interestingly, mitochondrial effects were not apparent in this dataset, although they have previously been implicated in larval survivorship and selection^21^.

We also show that certain gene ontologies confer bleaching tolerance (the overall phenotype) via host interactions with the symbionts. Five ontologies were significantly enriched in both the overall phenotype association and the symbiont proportion association, including peptide biosynthesis, Erk Cascade regulation and exopeptidase activity. ERK cascades phosphorylate hsp70^41^ and are central in extracellular signaling pathways, other components of which are also significantly enriched for high PBS values in this analysis (e.g., GTPase binding, Kinase activity, Phosphatase activity). However, only 10 of 40 loci (25%) and 5 of 28 gene ontologies (17%) shared significance between the association testing for binary phenotype and testing for proportion *Durusdinium*, supporting the hypothesis that host and symbionts contribute directly and indirectly to bleaching phenotype via a range of mechanisms.

These data also illustrate the biochemical consequences of functional variation which define bleaching phenotypes, including protein modification (i.e., protein phosphatase activity) and cell-signaling related to stress (i.e., ERK1/2 cascade). Interestingly, the molecular classes correlated with functional drivers of phenotype are decoupled from the metabolites which directly differentiate phenotype in a global analysis. For example, ref. ^34^ showed that DGCC lipids and steroids were disproportionately important for distinguishing phenotype and that fatty acids, monoacylglycerides, nucleotides, steroids and xanthins showed phenotype-specific responses to thermal stress^34^. None of these molecular classes are significantly enriched in the metabolite modules that correlate with gene ontologies defining phenotype or symbiont community in this study. We hypothesize that this difference reflects the host-specific regulation of physiology and biochemical patterns, rather than broader molecular patterns due to symbionts or the interaction of symbionts and the host. In particular, the conserved importance of protein kinases and phosphatases in biochemical patterns and thermally tolerant larval selection highlight the importance of this signaling mechanism in defining thermal tolerance. Although the correlations between GO loadings and metabolite module abundance were not significant after multiple comparisons correction, the congruent response of many molecules with several ontologies suggests that this is a biologically meaningful pattern, representing an important link that defines the downstream consequences of genomic variants besides eQTLs^37^.

There was also a link between two loci strongly predictive of bleaching phenotype and amino acid modifications and metabolism, potentially via Betaine Glycine (BG). Betaine Glycine is abundant in corals^42,43^, which have BG metabolism and biosynthesis pathways^44^, and can be found in *Montipora capitata* in Kāneʻohe Bay^42,45^. BG is an amino-acid osmolyte that has well-documented protective effects on plants during abiotic stress^46,47^ and is used in transgenic crop research^48^, where it may also counteract cellular signals that initiate HSP70 expression^49^. Ref. ^45^ also showed that osmolyte production, including BG, increased during temperature stress in *M. capitata*. Although the source of BG is unclear (i.e., host or symbiont^44^), the much higher abundances in bleaching resistant corals could represent a form of biochemical frontloading. This molecule is particularly important for the interaction of light and other abiotic stressors, including in corals^43^, which is closely aligned with the photosensitive nature of coral bleaching under high temperatures. Under these conditions, the repair of the D1 protein of photosystem II in symbionts is impaired, but the accumulation of BG prevents this obstruction and thereby limits oxidative stress. To the best of our knowledge, there is no known synthetic or metabolic connection between betaine glycine and the betaine lipids identified in ref. ^34^.

Symbiont communities are maternally inherited in *M. capitata*^50^ and appear fixed through time^30^, so BG variation between symbionts/phenotypes could be (a) due to genomic variation which impacts the ability of a genotype to harbor *Cladocopium* or *Durusdinium*, (b) due to differential symbiont physiology^34^ or (c) host plasticity induced by differential symbionts^51,52^. Another possibility is that cell-signaling (ERK/MAPK) implicated in both adult phenotype and larval selection analyses impact osmolyte cascades, which may play a role in bleaching^53^.

If host genomic variants drive this pattern, BG abundance could be influenced by selection not on variants in GDMT/SDMT itself, but some other regulatory machinery in close proximity, including PAX-C, or one of the methyltransferases, which could produce the same effects. We also observed a relative shift in the fitness of the site-wide cross between larval and juvenile stages. As larvae, the site-wide cross phenotype had slightly higher survivorship than pure nonbleached under heat stress, which could be an experimental artifact or an effect of increased genetic diversity in a cross with many more potential genetic combinations. After larvae settled and metamorphosed, this fitness advantage in the site-wide cross was lost and the pure nonbleached phenotype was significantly higher. We hypothesize that this decline is driven by the decreasing proportion of *Durusdinium* in the site-wide cross phenotype, which can be seen during the first 4 days of larval development in both ambient and high temperatures. This decline could be due to ‘hybridization’ between the two phenotypes which resulted in changing proportions of symbionts or differential selection due to heat stress; however, *Durusdinium* did not decrease in pure nonbleached corals with a small background of *Cladocopium*, supporting the explanation that the introduction of nonbleached eggs and bleached sperm lead to a decrease in fitness, regardless of temperature. This ‘underdominance’ response may occur in inter-specific hybridization experiments^54^ and warrants further examination of speciation and genetic differentiation in this system. An alternate hypothesis is that the breakdown of coadapted gene complexes had a delayed impact on phenotype which manifested after metamorphosis. It is also possible that fitness driven by the combination of paternal genes and symbiont leads to differential selection, which could be resolved in future experiments using indivudal crosses.

Selection on diverse host functional genes also occurred during larval development. We documented significantly enrichment of high PBS values in both cross and nonbleached phenotypes for cell-cell adhesion (GO:0022409), stress-activated protein kinase signaling cascades (GO:0031098) and ubiquitin-dependent protein catabolic processes (GO:0043162), which reflect major functions that are impacted regardless of background genetics and correspond to well-known heat-stress responses in corals^40^. Signaling cascades were also implicated in differentiation of adult phenotypes, supporting the role of the cellular processes that govern interactions betwen the host and symbionts or coordinate gene complexes in response to heat stress^55,56^.

Importantly, less than 10% of significant ontologies in this anlaysis were shared between the two phenotypes, indicating differential selection trajectories. These differences may be due to epistatic effects arising from the polygenic nature of thermal tolerance that vary between phenotypes or the influence of the symbiont communities on larvae. Notably, a broader array of loci and gene ontologies are selected in cross corals and they are selected more strongly, indicating that thermally tolerant corals may be ‘preadapted’ to dealing with some thermal stress, corresponding closely with ref. ^13^. The strong nonbleached-specific selection of twenty functional categories supports this hypothesis and, although we cannot evalute classical selective breeding approaches based on host genetics, emphasizes the importance of host heritability underlying thermal tolerance. If symbiont and genomic context are indeed important for selection trajectories, the outcomes of adaptation of vertically transmitting corals under climate change may be significantly more complex than those of horizontal spawning corals.

The role of different symbionts in defining thermal tolerance has been documented in many studies in adults, but most corals have azooxanthellate larvae, so the role of infection (or transmission) of different symbionts in the larval stage remains poorly defined^57^, although these differences impact physiology^58^ and can dramatically alter host gene expression^51,52^. Juvenile *Acropora spathulata* infected with *Durusdinium trenchii* bleached less than those infected with other symbionts^59^, especially when one or both parents were from warm-adapted reefs, which closely mirrors survivorship patterns in juveniles in our study, assuming the *Cladocopium:Durusdinium* ratios found in larvae were maintained. While this system does not allow us to isolate host effects, the phenotypic variance explained by genotype and selection against *Durusdinium* in larvae strongly suggests that host and symbiont affects both impact thermal tolerance.

The data presented here document the synergistic impact of host genome and symbiont community on coral phenotype in the absence of differentiating selection. This variance is the raw material upon which adaptation under climate change will act if previously bleached corals are the first to succumb to repeated bleaching events. Our data also suggest that the raw materials for adaptive recombination need not occur over large environmental gradients and that symbiosis ecology and demographic processes can generate this variance on individual reefs.

## Methods

### Collections

In 2015, the summer thermal maximum produced a mosaic of bleaching in Kāneʻohe Bay, where adjacent colonies either severely bleached or remained visually healthy^29,60,61^. The majority of these colonies recovered and have been tracked through time (Supplementary Fig. 1). We selected 22 healthy colonies (11 pairs) along a 2–3 m depth contour on Reef 13 in Kāneʻohe Bay and used them for genetic, metabolomic and gamete collections for selective breeding. We collected gamete bundles from each colony in situ on 13 July 2018 and re-collected 3 replicate branches for adult genetics and symbiont extractions in August 2018 (to avoid spawning impacts). We collected metabolite samples from each colony in May-September 2019^34^. To examine species boundaries in the context of our phenotypes, we also collected 3 fragments of *Montipora flabellata* from northern Kāneʻohe Bay in July 2021. Adult fragments were stored at −20 °C until processing.

### Crosses, fertilization and treatments

All 22 colonies in the study spawned on 13 July 2018. We created a site-wide cross (gametes from all-colonies in the study), bleached phenotype cross (only gametes from historically bleached parents) and nonbleached phenotype cross (only gametes from historically nonbleached parents) prior to the breakup of bundles and fertilization (Supplementary Fig. 1). Gamete bundles from each bulk cross were immediately aliquoted (*n* = 10) for fertilization^62^ and returned to the Hawaii Institute of Marine Biology. Triplicates of fertilized embryos from each phenotype were added to one 150 L larval rearing conical and allowed to develop for 12 h to the prawn-chip stage before temperature treatments (ambient: 27.4 ± 0.55 °C; mean ± 1 SD and high: 30.2 ± 0.51 °C) began for each of three phenotype pools (Supplementary Fig. 2).

### Larval Survivorship, Juvenile Settlement and Survivorship

We established replicate survivorship trials at 12 h post fertilization (hpf; *n* = 15 per phenotype and temperature) and measured larval survivorship at 27, 47, 75, 96, 120, 172 and 213 (hpf) (Supplementary Fig. 2). At 109hpf, larvae from each phenotype at ambient temperatures were transferred to settlement chambers (*n* = 4) and allowed to settle on pre-conditioned aragonite plugs. After 8 days exposure to substrate, plugs (*n* = 877) were randomly allocated into ambient (27.6 ± 0.96 °C) and high (30.1 ± 0.38 °C) temperature treatments (*n* = 2 per temperature) (Supplementary Fig. 3).

Settlement plugs were photographed at the initiation of temperature treatments (12 days post fertilization) and at 8, 14, 18, 27 and 34 days in treatments. Individual settlers were counted from photos at each timepoint. The removal of larvae for settlement did not interrupt the ongoing larval survivorship trials. See Supplementary Fig. 2 for details.

Larval and juvenile survivorship was analyzed with Kaplan-Meier regression in the R package *Survival*, with pairwise comparisons between temperature and phenotype crosses using a likelihood ratio test (implemented by *Survminer*::*pairwise_survdiff*) with Bonferroni adjusted p-values. We used a generalized linear model examining final survivorship (averaged per replicate or plug) with temperature and phenotype as fixed effects and a quasibinomial family. We calculated broad-sense heritability of both larvae and juveniles (excluding the site-wide cross) separately at each temperature as variance (MSE) explained by Phenotype/total variance (MSE) in a 1-way ANOVA, modified from ref. ^63^.

### Symbiont analysis

Larval symbiont collections were made at 16, 36, 60 and 84 h post fertilization, representing daily sampling that overlapped genomics sampling (Supplementary Fig. 2). Three replicates of 30–50 larvae were haphazardly collected from each treatment. Adult fragments and larval time-series collections were extracted with a CTAB-chloroform protocol (following^22^). Concentration of *Cladocopium, Durusdinium* and the coral host were quantified via quantitative PCR using actin and Pax-C assays^29,64,65^ on a StepOnePlus system (Applied Biosystems) with two technical replicates. Samples were re-extracted and analyzed if C_T_ replicate standard deviation was greater than 1 between replicates or if only a single replicate amplified. C_T_ values were corrected for fluorescence and copy number using the equations of ref. ^29^. See Supplementary Fig. 2 for details.

### Library preparation and sequencing

Larval samples were collected from each conical (*n* = 6) at 16 hpf by collecting 5 replicates of 50 larvae (*n* = 250 total larvae per timepoint per treatment), preserved in 2% SDS in DNAB and stored at −20 °C until processing. A second sample was collected in the same way at 84 hpf, representing timepoints near the initiation of heat stress during late development and after the putative selective pressure had led to ~25% mortality in high treatments (Supplementary Fig. 2).

DNA was extracted using a DNeasy Blood & Tissue Kit (Qiagen) following manufacturer protocol. Extracted DNA was quantified with a Qubit fluorometer and 300 ng of DNA was digested with *HindIII* (New England Biolabs). Libraries (*n* = 23; 22 adult samples and an adult technical replicate, 60 larval samples) were prepared with an Illumina TruSeq Nano kit and TruSeq adapters for 350 bp inserts following manufacturer protocol. Samples were sequenced on a single lane of an Illumina HiSeq 4000 with paired end 150 bp reads (Genewiz, South Plainfield, NJ). To examine species boundaries, we also sequenced 3 samples of *Montipora flabellata* from Kāneʻohe Bay as part of a separate sequencing project (Illumina WGS, paired end 250 bp reads, Illumina NovaSeq 6000).

We prepared a reference genome by mapping the *Montipora capitata* genome to the chromosome scale scaffolding of *Acropora millepora*^20^ using default options in RagTag^66^, which resulted in ~5% higher alignment rates and higher contiguity. The *Montipora capitata* individual used for genomic sequencing showed signals of gene duplication^67^ and is ~885MB (compared to 458 MB for the *A. millepora* reference). We included un-anchored *M. capitata* sequences as additional contigs, effectively increasing the contiguity of the reference without removing any data. Short read alignment rates were substantially lower against the original *A. millepora* genome or against the chromosome scale *M. capitata* anchored alignment without additional contigs. We annotated predicted protein sequences from the *M. capitata* assembly^67^ using blastp against Uniprot/Swissprot and extracted gene ontologies using a custom script. We used annotations from the *A. millepora* assembly^20^ for scaffolded contigs and the additional annotations for unscaffolded *M. capitata* sequences using a custom R script.

### Adult population genetics

Raw reads were demultiplexed at the sequencing facility and we trimmed adapters using *cutadapt*^68^ before aligning all samples using *bwa mem*^69^. We analyzed adult clonality with *M. flabellata* samples using a biological replicate in *ANGSD*^70^ to generate an identity by state matrix with filtered reads (minMapQ 30, minQ 30) and used hierarchical clustering to confirm species assignments and that there were no clonemates in our adult population. This analysis does not adhere to an evolutionary model (e.g., Ts/Tv rates or nucleotide frequencies) and therefore should not be interpreted as a phylogeny. We calculated F_ST_ between phenotypes from site frequency spectrums using *ANGSD*/*realSFS*. We used *NGSadmix*^71^ (K = 1, K = 2) to examine population structure with default settings. We examined population structure using principal components analysis using the predicted number of secondary alleles^3^ for each loci in each sample as input data. We examined variance explained by phenotype using a PERMANOVA in the R package *vegan*^72^.

### Adult associations with phenotype and symbiont state

We used two association comparisons to distinguish (1) host effects on binary phenotype, (2) host-symbiont interactions free of phenotype assumptions. First, we examined the association of individual loci with each adult phenotype (*n* = 21, one sample removed with low depth) using genotype probabilities in ANGSD (GL 2, doAsso 1, doGeno 8, doPost 1, minDepth 5, SNP *p* value <2e^−6^) and a case-control framework for binary phenotype assignments. We permuted phenotype assignments between colonies 10,000 times, recalculated association metrics and selected the highest LRT value for any individual loci in each permutation. We assigned the 99.99^th-^percentile of this LRT distribution as the cutoff for significance (*p* < 0.0001) for individual loci following^20^.

To differentiate host-symbiont interactions from background genetic structure and phenotype assumptions, we reconducted the association analysis above (ANGSD GL 2, doAsso 6, doGeno 8, doPost 1, minDepth 5, SNP *p* value < 2e^−6^, minHigh 3, minCount 3) using mean proportion of *Durusdinium* (see Fig. 1c) as the dependent variable in a logistic regression. This analysis takes advantage of intermediate phenotypes and the individuals that do not agree with the overall phenotype-symbiont patterns.

For both comparisons, we used annotations within 2.5kbp of each variant to derive ontologies for two-tailed GO_MWU^73^. This strategy ranks individual genes by ‘heat’ and then resolves gene ontologies with at least 15 representative genes that ranked significantly (FDR *p* < 0.1) higher or lower than background expectations in representing functional groupings that differ based on the comparison. We treated the LRT statistic from the binary association as ‘heats’ and reconducted this analysis for associations with symbiont state using the effect size (beta) andthe LRT. We extracted all loci annotated with each ontology that significantly described binary phenotype and ran a *PCAngsd* subroutine with these loci to summarize genetic differentiation from all adult colonies in each functional category.

### Larval analysis

To evaluate selection in the larval resequencing experiment, we created alignment files of pooled larval replicates (*n* = 5) by treatment (*n* = 2), phenotype (*n* = 3) and timepoint (*n* = 2) and pooled adults by phenotype (see Supplementary Table 1 for details). We summarized allele counts using *bcftools*^74^ and exported biallelic variants. There was low and uneven coverage in the bleached samples, so we excluded this pool from downstream analysis. We analyzed per-site F_ST_ for each sample (depth > =10) using the R package *poolfstat*^75^ and calculated a Population Branch Statistic (PBS)^76^ for each phenotype (see Supplementary Table 1 for details). To ask how heat selection impacted specific loci, we constructed a PBS statistic distinguishing the final post-heated larval pool from the initial larval pool and the final ambient larval pool (see Supplementary Table 1 for details) for each phenotype.

In this context, the PBS statistic is ideal for low-depth data because large values are derived from cases where allele frequencies are similar in both the final ambient pool and the initial heated larval pool. We also filtered the dataset to loci where both alleles were found in parental and initial pools and where the initial-to-parental F_ST_ was less than 0.05, assuring that signals of heterozygosity were not a sequencing artifact. PBS of the comparison of interest is also positively influenced by similarity in the ‘outgroups’, in this case the final ambient larval pool and the initial heated pool, which isolates the effect of the heat treatment. This expected pattern (based on random crosses from the same pool) further supports consistent sequencing outcomes, minimizes artifacts and highlights selection due to temperature stress.

We compared the PBS statistic for all loci shared between the cross and nonbleached phenotype with a linear regression and a Wilcox test. To evaluate selection for specific functions, we used GO_MWU^73^, treating the PBS statistic as ‘heats’ to look for gene ontologies enriched in large PBS values, which denote heat-specific selection. After conducting this analysis on each phenotype, we selected only significant ontologies (FDR *p* < 0.1) and examined shared ontologies between phenotypes.

We then calculated the difference in PBS between each phenotype and re-ran the GO_MWU analysis. This approach yields larger values when selection in the nonbleached pool was stronger than the cross pool, isolating functional categories that are disproportionately selected in nonbleached corals and contribute to selective breeding outcomes.

### Metabolite analysis

Small biopsies of all 22 parent corals were extracted in 500uL 70% methanol, kept on ice for 30 min and stored at −80 °C until shipping to Michigan State University. Methanolic extracts were analyzed using LC-MS/MS. Details of chromatography and mass spectrometry parameters can be found in ref. ^34^. The converted.mzXML files were processed with MZmine2^77^ and the feature quantification table was exported and processed with the Global Natural Products Social Molecular Networking (GNPS)^78,79^ and CANOPUS on SIRIUS 4^80,81^. MZmine2 parameters for processing mass spectrometry data are described in^34^, and the CANOPUS parameters can be found in Supplementary Table 2. Both CANOPUS output files (“canopus_summary.csv”, “compound_identification.csv”) were merged and filtered to include the most specific class of molecules, matches and their molecular formulas. Molecular features from blanks and found between both files that did not display in silico annotations from CANOPUS and correlation between molecular formulas were removed.

We repurposed the WGCNA analytical framework for metabolites, which summarizes molecules with highly correlated abundances into modules^82^. We used log2 transformed abundances after imputing missing data with the lowest abundance of each molecule following^83,84^. We tested a power threshold from 1 to 20 and chose 6 as the ‘elbow’ in scale-free topology, with modules merged >0.15, minKME = 0.8, at least 25 molecules per module and an unsigned network. We used the first principal component from *PCAngsd* as a summary of the genetic variation of each coral’s genes relating to that ontology as the ‘trait’ for Spearman correlation analysis with WGCNA. We used a Fisher’s exact test to test for enrichment in molecular families within each module and calculated a false discovery rate-corrected p-value.

### Betaine analysis

After noting the high association and strong linkage between two loci with high association values (*p* < 0.0001), we extracted a 50 kb window centered at the midpoint of these loci and annotated with blastn against cnidarians. Among other genes, we documented glycine sarcosine dimethylglycine N-methyltransferase within this selection window, a gene family that produces Betaine Glycine (BG), which we annotated in the metabolite data (metabolomics standards initiative level two^85^). We compared Betaine relative abundances between phenotypes using a Wilcox test. We used the raw annotations from GO_MWU (before merging into the parent term ‘Cellular Modified Amino Acid Metabolic Process’), and compared the association values for ‘Amino-Acid Betaine Metabolic Process’ to all other loci using a Wilcox test. We compared the beta values for predicting proportion Durusdinium in this window with the rest of the genome using a Wilcox test.

### Reporting summary

Further information on research design is available in the Nature Research Reporting Summary linked to this article.

## Supplementary information

Supplemental Material

Reporting Summary

## Data Availability

All analysis scripts, ecological data and processed sequencing data are available at github.com/druryc/mcap_bleaching. Raw sequencing data is available at NCBI PRJNA597077. Feature-based molecular networking is available at: https://gnps.ucsd.edu/ProteoSAFe/status.jsp?task=bb9b6126118c4ba1881f4483560012d3 and raw files are available at massive.ucsd.edu under MassIVE ID MSV000085272 and MSV000085925.
