# Supplementary material for: Intrapopulation adaptive variance supports thermal tolerance in a reef-building coral
Source: Commun Biol. 2022 May 19;5:486. doi: 10.1038/s42003-022-03428-3 (PMC9120509; doi:10.1038/s42003-022-03428-3)
Supplement: Supplementary file 1 — Supplemental Material [file 42003_2022_3428_MOESM1_ESM.pdf]

Supplementary information for:

Intrapopulation adaptive variance supports thermal tolerance in a reef-building coral

Crawford Drury[1], Nina K. Bean[1‡], Casey I. Harris[1‡], Joshua R. Hancock[1], Joel Huckeba[1,2], Christian Martin H[3], Ty Roach[1], Robert Quinn[3], Ruth D Gates[1]

1 Hawaiʻi Institute of Marine Biology, University of Hawaiʻi, Kāneʻohe, HI, USA
2 University of Amsterdam, Amsterdam, Netherlands
3 Department of Biochemistry and Molecular Biology, Michigan State University, East Lansing, MI, USA

[‡]these authors contributed equally
Correspondence: crawford.drury@gmail.com

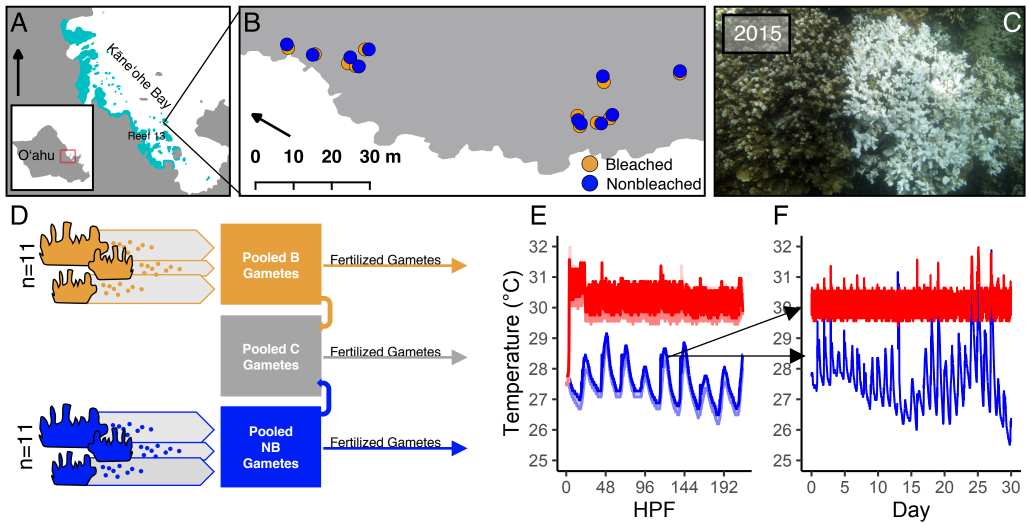

Supplementary Figure 1 – Site and Experimental Framework

A) Gamete collections were made at Reef 13 in Kāneʻohe Bay, Oʻahu, Hawaiʻi on 13 July 2018. B) Individual colonies were netted for collections using C) pairs identified in the 2015 bleaching event to minimize microhabitat differences and select for parental thermal tolerance. D) Gametes were collected from 11 colonies of each phenotype and used to create pools of all nonbleached gametes and all bleached gametes. The site-wide cross was then created from equal volumes of the bleached and nonbleached pools and all three pools were exposed to E) larval temperature treatments downstream starting 12 hours after fertilization. At 109 hours after fertilization, larvae from all three phenotypes were allowed to settle on preconditioned aragonite plugs for 8 days and then exposed to F) juvenile temperature treatments. See Supplementary Fig. 1-2 for full details. Color scheme maintained in subsequent figures.

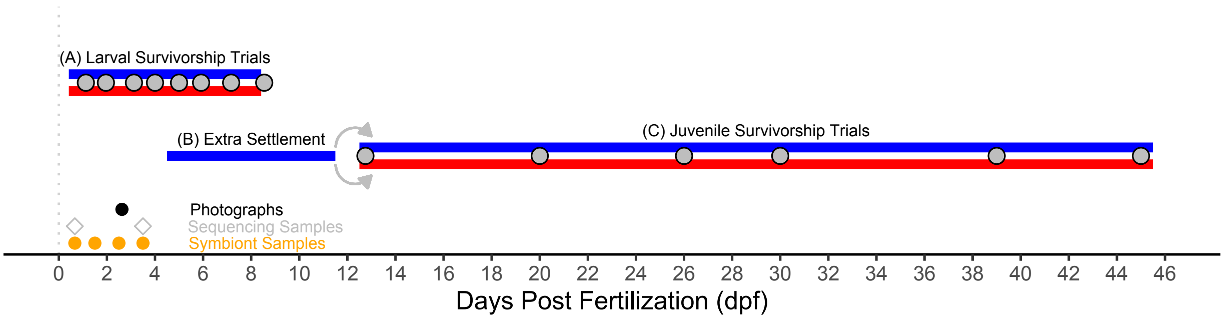

Supplementary Figure 2 - Experimental Timeline

Timeline in days post fertilization detailing temperature treatments, sequence, and data collection. Blue and red bars represent duration of treatment for larval and juvenile stress tests, with nested gray dots at survivorship survey timepoints. Points correspond to data collection for symbiont samples, larval sequencing and photographs for growth measurements. Briefly, (A) larvae were aliquoted into 50mL tubes for survivorship analysis at two temperatures on 1 dpf. Sequencing samples were collected from larval cultures (not from survivorship aliquots) at 1 and 4 dpf. (B) After final sequencing samples were collected (4 dpf) extra larvae at ambient temperatures were allowed to settle at ambient temperatures without interrupting survivorship aliquots. (C) These settled juveniles were randomly allocated to high and ambient temperatures at 12 dpf and monitored until 45dpf.

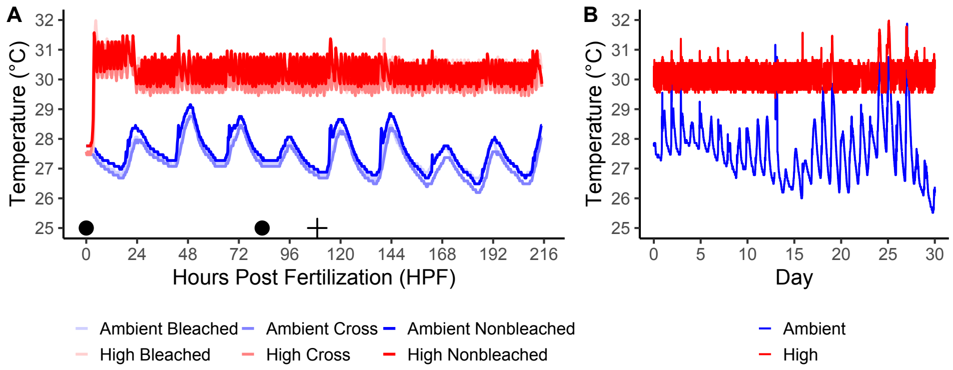

Supplementary Figure 3 – Temperature Profiles

A) Temperature profiles from each conical during the larval phase. Black circles denote timing of genetics sampling. Cross denotes timing of transfer of remnant ambient larvae to settlement chambers. B) Temperature profiles from each conical during the juvenile phase. Colors correspond to temperature treatment (blue=Ambient, red=High).

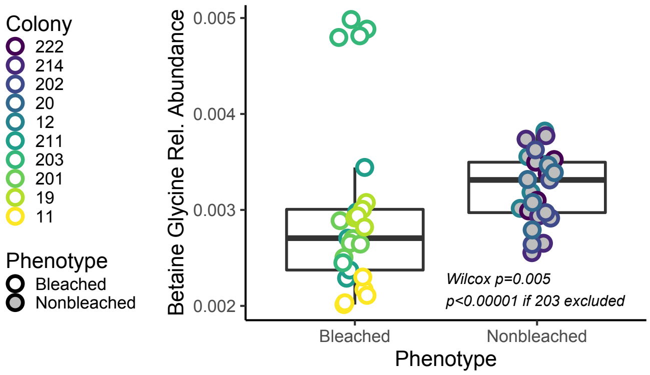

Supplementary Figure 4 – Betaine Glycine Abundances

Betaine Glycine abundances from parent colonies in this study.

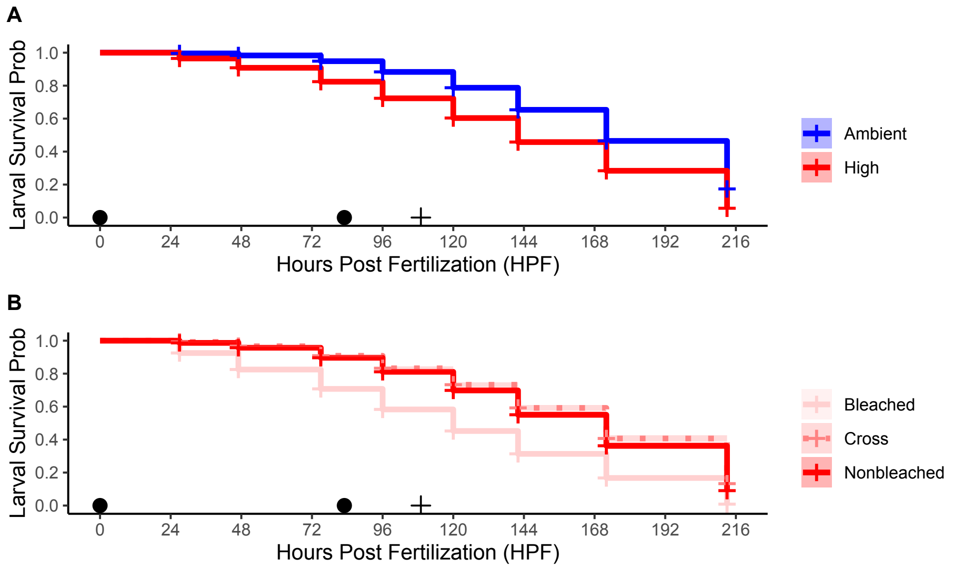

Supplementary Figure 5 – Survivorship Dynamics of Larval *M. capitata*

A) Probability estimates from Kaplan-Meier larval survivorship fits for each temperature over hours in temperature treatment, line shown with 95% confidence interval shading. B) Probability estimates from Kaplan-Meier larval survivorship fits for each phenotype in high temperature treatment, line is shown with 95% confidence interval shading. Black circles denote timing of genetics sampling. Plus denotes timing of transfer of remnant ambient larvae to settlement chambers. Colors correspond to temperature treatment (blue=Ambient, red=High), transparency corresponds to phenotype.

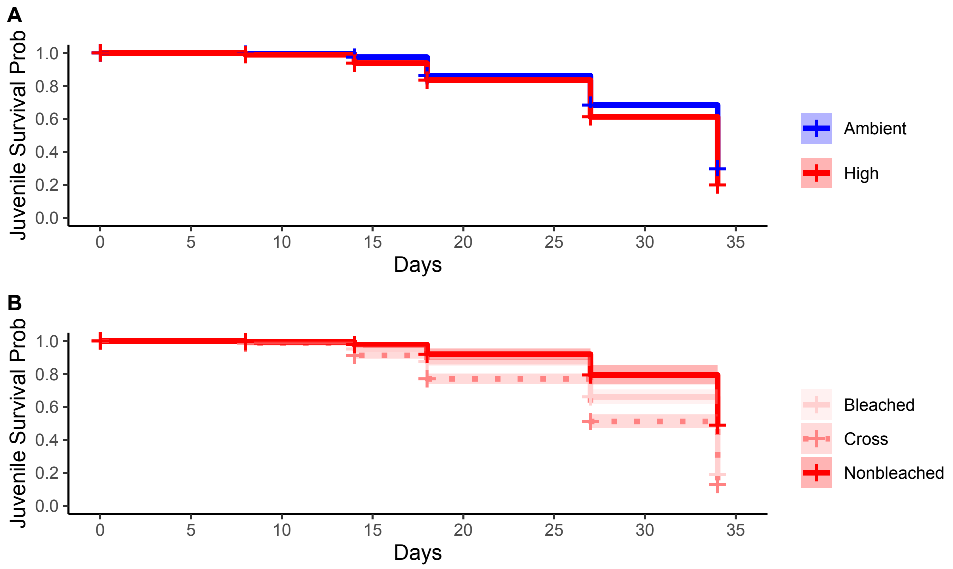

Supplementary Figure 6 – Survivorship Dynamics of Juvenile *M. capitata*
A) Probability estimates from Kaplan-Meier juvenile survivorship fits for each temperature over hours in temperature treatment, line shown with 95% confidence interval shading. B) Probability estimates from Kaplan-Meier larval survivorship fits for each phenotype in high temperature treatment, line is shown with 95% confidence interval shading. Black circles denote timing of genetics sampling. Plus denotes timing of transfer of remnant ambient larvae to settlement chambers. Colors correspond to temperature treatment (blue=Ambient, red=High), transparency corresponds to phenotype.

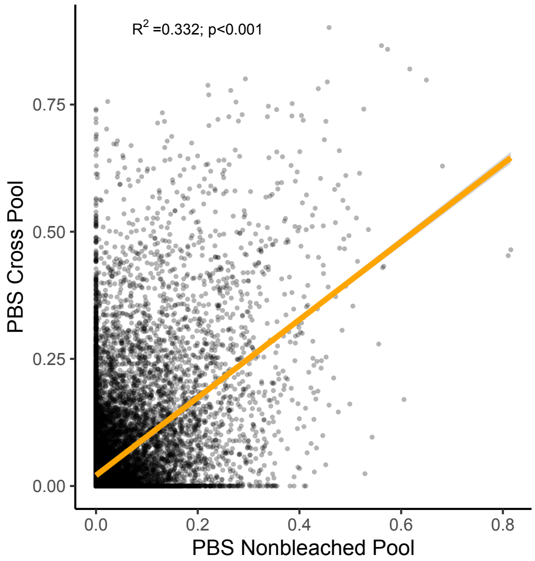

Supplementary Figure 7 – PBS values for the cross and nonbleached pools

PBS statistics were calculated separately for each loci in the cross and nonbleached pools using the framework in Supplemental Table 1. Briefly, we calculated the PBS statistic for each phenotype (nonbleached, site-wide cross) separately, distinguishing the final post-heat larval pool from the initial heated larval pool and the final ambient larval pool. Orange line represents linear regression.

Supplementary Table 1

PBS calculation framework, comparing heat-selected larval pools of each phenotype to initial and parental pools. T= $-\log(1-F_{ST})$, where pairwise F_{ST} was calculated on a per-site basis for each variant (i.e., T_{FA} = T based on pairwise F_{ST} between outgroup A and the focal group.)

Phenotype	(F) Focal Group	(A) Outgroup A	(B) Outgroup B	Formula
NB	Final High Pool (n=250)	Initial High Pool (n=250)	Final Ambient Pool (n=250)	PBS = $(T_{FA} + T_{FB} - T_{AB})/2$
Cross	Final High Pool (n=250)	Initial High Pool (n=250)	Final Ambient Pool (n=250)	PBS = $(T_{FA} + T_{FB} - T_{AB})/2$

Supplementary Table 2

SIRIUS 4 parameters used for the *in silico* molecular formula annotations of the mass spectrometry data generated from MZmine 2. Mascot graphical format file (mgf) was imported and processed as follows:

Module	Parameters	
Sirius	Instrument	Orbitrap
	MS^2 isotope scorer:	Score
	MS^2 mass deviation	5 ppm
	Candidates	5
	Databases	Bio database, GNPS, Natural Products
ZODIAC	Default	
CSI: FingerID	Databases	Bio database, GNPS, Natural Products
	Adducts	$M+H]^+$, $[M+Na]^+$ and $[M+K]^+$
	Enforce	True
CANOPUS	True	

Supplementary Table 3

Annotations from individual genes significantly (p<0.0001) associated with bleaching phenotype in adults. Black text denotes annotations from megablast, blue text denotes annotations from discontinuous megablast.

locus	gene name	e-value	organism	function	ref
114:270077-1166526(+)_382645	Ethanolamine phosphotransferase 1-like	2e-64	*Acropora digitifera*	Sphingolipid biosynthesis	25
1142:22067-267875(+)_240011	Ankyrin repeat and zinc-finger domain-containing protein 1-like	2e-12	*Acropora digitifera*	Immunity	1,32
135:165149-681565(+)_107553	Monocarboxylate transporter 10-like	1e-124	*Acropora millepora*	Nitrogen cycling; amino acid metabolism	2
16:103141-1930322(+)_88237	Histamine H2 receptor-like	3e-71	*Acropora millepora*	Immune response	3
175:84843-855127(+)_390354	PAX-C	4e-114	*Montipora sp.*	Growth/development; host-algal symbiosis regulation	4,5
23:0-1877333(+)_1275116	Neuropeptide SIFamide receptor	8e-37	*Orbicella faveolata*	Larval migration, settlement, metamorphosis	6,7
2374_33003	18s rRNA	0.0	*Montipora verrucosa*	Protein synthesis	28
2644:0-29873(+)_8082	Histamine N-methyltransferase-like	0.0	*Acropora millepora*	Methylation (humans)	8
316:172135-793760(+)_405758	PAX-C	6e-83	*Montipora solanderi*	Growth/development, host-algal symbiosis regulation	4,5
32:221614-1703322(+)_986481	Histamine H2 receptor-like	2e-38	*Acropora millepora*	Immune response	3
362:154151-476426(+)_296306	Unknown	2e-24	*Acropora millepora*	Unknown	-
416:25677-496747(+)_417623	Mucin-like	2e-31	*Acropora digitifera*	Mucus generation/secretion; DNA damage repair	9,23
462:90630-579403(+)_429238	Octopamine receptor beta-1R-like	3e-73	*Orbicella faveolata*	Oocyte development/maturation	10

5:218022-2430108(+)_851904	Adenosine receptor A2a-like	2e-93	*Acropora millepora*	Immune response; inflammation	1,11
50:672149-1522693(+)_465625	Polyubiquitin-C	0.0	*Stylophora pistillata*	Oxidative stress resistance; protein catabolism	12,29
553:474523-500157(+)_13812	Ribonuclease Y-like	7e-133	*Orbicella faveolata*	Unfolded protein response	13
615:456522-469093(+)_1880	Plexin-B-like	1e-47	*Acropora millepora*	Cytoskeleton dynamics; cell adhesion; axon guidance	14,23
69:44661-1355009(+)_72740	Phosphoinositide 3-kinase regulatory subunit 4-like	2e-58	*Acropora digitifera*	Host autophagy	15
94:79413-1187499(+)_1064347	kelch-like protein diablo	8e-113	*Acropora millepora*	Oxidative stress response; protein ubiquination	26, 30
chr1_RagTag_22018874	Golgi-associated PDZ and coiled-coil motif-containing protein-like	8e-77	*Acropora millepora*	Protein binding/transport	16
chr1_RagTag_31003888	Dynein assembly factor 3, axonemal-like	2e-32	*Actinia tenebrosa*	Cytoskeletal construction; autophagy	17,31
chr10_RagTag_8284035	Putative ankyrin repeat protein RF_0381	1e-49	*Dendronephthya gigantea*	Immunity	1
chr11_RagTag_4203672	Neurexin	2e-68	*Acropora digitifera*	Biomineralization	18
chr11_RagTag_4215008	TFIID subunit 5-like	1e-46	*Acropora digitifera*	Cell signaling; transcription factor activity; protein binding	21
chr12_RagTag_25257908	Phosphoinositide 3-kinase regulatory subunit 4-like	5e-65	*Acropora digitifera*	Host autophagy	15
chr2_RagTag_20154540	Tetratricopeptide repeat protein 28	2e-108	*Orbicella faveolata*	Apoptotic signaling	27
chr4_RagTag_11047240	kelch-like protein diablo	6e-45	*Acropora digitifera*	Oxidative stress response; protein ubiquination	26,30

chr4_RagTag_5271861	Unknown	0.0	*Acropora millepora*	Unknown	-
chr5_RagTag_11207344	Unknown	0.0	*Acropora millepora*	Unknown	-
chr6_RagTag_13411819	Arginine/serine-rich protein PNISR-like	8e-145	*Acropora digitifera*	Protein synthesis	19
chr8_RagTag_12409112	Neurexin	6e-70	*Acropora digitifera*	Biomineralization	18
chr8_RagTag_4922595	Ankyrin repeat and zinc-finger domain-containing protein 1-like	2e-12	*Acropora millepora*	Immunity	1,32
Sc0000015_RagTag_1507093	E3 ubiquitin-protein ligase MARCH5-like	2e-58	*Acropora millepora*	*Symbiodiniaceae* stress response; protein degradation	20,21
Sc0000134_RagTag_502413	Somatostatin receptor type 4-like	3e-160	*Acropora millepora*	Myoregulatory activity; neural pathways; MAP kinase and adenylate cyclase interactions	22
Sc0000201_RagTag_77657	D-inositol 3-phosphate glycosyltransferase-like	4e-50	*Acropora digitifera*	Host/algal symbiosis regulation	23
Sc0000204_RagTag_980637	Plexin-B-like	1e-64	*Acropora millepora*	Cytoskeleton dynamics; cell adhesion; axon guidance	14,23
xpSc0000570_RagTag_25400	Cationic amino acid transporter 1-like	2e-58	*Stylophora pistillata*	Protein/membrane formation	24

Supplementary Table 4

Summary of Gene Ontologies describing differences in binary phenotype. Listed Gene Ontologies are significantly enriched in high LRT values from the phenotype association analysis, suggesting functions that are significantly different between adult phenotypes and distinguish bleaching tolerance.

Term	Name	P (fdr)	Category
GO:0006412	translation	0.084	BP
GO:0006476; GO:0035601; GO:0098732	protein deacetylation	0.028	BP
GO:0006518	peptide metabolic process	0.046	BP
GO:0006575	cellular modified amino acid metabolic process	0.045	BP
GO:0018200	peptidyl-glutamic acid modification	0.028	BP
GO:0034334; GO:0034332	adherens junction organization	0.045	BP
GO:0043043	peptide biosynthetic process	0.059	BP
GO:0043603	cellular amide metabolic process	0.085	BP
GO:0070373	negative regulation of ERK1 and ERK2 cascade	0.048	BP
GO:0071333; GO:0009749; GO:0071331; GO:0009746; GO:0034284; GO:0009743; GO:0071326; GO:0071322	cellular response to hexose stimulus	0.077	BP
GO:0090630	activation of GTPase activity	0.085	BP
GO:1901565	organonitrogen compound catabolic process	0.077	BP
GO:1902414	protein localization to cell junction	0.024	BP
GO:1903614; GO:1903613; GO:1904894; GO:0018279; GO:0006487; GO:0018196	protein N-linked glycosylation	0.027	BP
GO:0031902	late endosome membrane	0.099	CC
GO:0000287	magnesium ion binding	0.094	MF
GO:0003735	structural constituent of ribosome	0.094	MF
GO:0003887; GO:0004523; GO:0016891	DNA-directed DNA polymerase activity	0.094	MF
GO:0004177; GO:0008238;	exopeptidase activity	0.094	MF

GO:0070006; GO:0008235			
GO:0004190; GO:0070001	aspartic-type endopeptidase activity	0.094	MF
GO:0008138	protein tyrosine/serine/threonine phosphatase activity	0.093	MF
GO:0008199; GO:0004322; GO:0016724; GO:0016722	ferric iron binding	0.094	MF
GO:0016758; GO:0016757	transferase activity, transferring glycosyl groups	0.093	MF
GO:0017016; GO:0031267	small GTPase binding	0.094	MF
GO:0017048	Rho GTPase binding	0.094	MF
GO:0030144; GO:0140103; GO:0030145; GO:0004864; GO:0019212	protein phosphatase inhibitor activity	0.093	MF
GO:0051020	GTPase binding	0.028	MF
GO:0070851; GO:0005138	growth factor receptor binding	0.094	MF

Supplementary Table 5

Summary of Gene Ontologies describing similarities between binary phenotype. Listed Gene Ontologies are significantly enriched in low LRT values from the phenotype association analysis, suggesting functions that are significantly similar between adult phenotypes

Term	Name	P (fdr)	Category
GO:0001667	ameboidal-type cell migration	0.048	BP
GO:0001932; GO:0042325	regulation of phosphorylation	0.037	BP
GO:0002252; GO:0051607; GO:0009615	immune effector process	0.045	BP
GO:0002376	immune system process	0.077	BP
GO:0008037	cell recognition	0.077	BP
GO:0008202	steroid metabolic process	0.006	BP
GO:0009605	response to external stimulus	0.077	BP
GO:0009888	tissue development	0.070	BP
GO:0009952	anterior/posterior pattern specification	0.051	BP
GO:0010035	response to inorganic substance	0.045	BP
GO:0010038	response to metal ion	0.084	BP
GO:0010557; GO:0010628; GO:0031328; GO:0009891	positive regulation of gene expression	0.054	BP
GO:0010638	positive regulation of organelle organization	0.048	BP
GO:0016192	vesicle-mediated transport	0.027	BP
GO:0019827; GO:0098727	stem cell population maintenance	0.048	BP
GO:0022604	regulation of cell morphogenesis	0.056	BP
GO:0030278	regulation of ossification	0.077	BP
GO:0030517	negative regulation of axon extension	0.095	BP
GO:0031122	cytoplasmic microtubule organization	0.070	BP
GO:0043207; GO:0009607; GO:0051707	response to biotic stimulus	0.029	BP
GO:0045087	innate immune response	0.045	BP
GO:0045596	negative regulation of cell differentiation	0.044	BP
GO:0045597	positive regulation of cell differentiation	0.092	BP
GO:0045859; GO:0043549; GO:0051338	regulation of transferase activity	0.008	BP
GO:0045926	negative regulation of growth	0.083	BP
GO:0045944	positive regulation of transcription by RNA polymerase II	0.045	BP

GO:0050685	positive regulation of mRNA processing	0.085	BP
GO:0050793	regulation of developmental process	0.056	BP
GO:0051093	negative regulation of developmental process	0.024	BP
GO:0051094	positive regulation of developmental process	0.070	BP
GO:0051128	regulation of cellular component organization	0.020	BP
GO:0051130	positive regulation of cellular component organization	0.028	BP
GO:0051240	positive regulation of multicellular organismal process	0.026	BP
GO:0051241	negative regulation of multicellular organismal process	0.027	BP
GO:0051704	multi-organism process	0.045	BP
GO:0051965; GO:0051963	regulation of synapse assembly	0.085	BP
GO:0070988	demethylation	0.029	BP
GO:0070989	oxidative demethylation	0.006	BP
GO:0071360; GO:0071359; GO:0009597; GO:1900246; GO:0039535; GO:0039531; GO:0032481	cellular response to dsRNA	0.024	BP
GO:0090068; GO:0045787	positive regulation of cell cycle	0.084	BP
GO:0005783	endoplasmic reticulum	0.099	CC
GO:0048786	presynaptic active zone	0.099	CC
GO:1990909; GO:1990851	Wnt signalosome	0.099	CC
GO:0005509	calcium ion binding	0.094	MF
GO:0008395; GO:0070330; GO:0016712; GO:0032451; GO:0101020; GO:0008401; GO:0050649	aromatase activity	0.009	MF
GO:0015026; GO:1904928; GO:0071936; GO:0042813	coreceptor activity	0.094	MF
GO:0020037; GO:0046906	tetrapyrrole binding	0.094	MF
GO:1990837; GO:0003690; GO:0043565	double-stranded DNA binding	0.093	MF

Supplementary Table 6

Summary of Gene Ontologies describing proportion *Durusdinium*. Listed Gene Ontologies are significantly enriched in high LRT or effect size values from the symbiont association analysis, suggesting functions that are significantly related to symbiont community.

Term	Name	P (fdr)	Category
GO:0043604	amide biosynthetic process	0.053	BP
GO:0048468	cell development	0.054	BP
GO:0043161; GO:0010498; GO:0030163; GO:0051603; GO:0006511; GO:0019941; GO:0043632; GO:0044265	cellular macromolecule catabolic process	0.098	BP
GO:0032774; GO:0009059; GO:0034654; GO:0018130; GO:0019438; GO:0044271; GO:1901362; GO:0006351; GO:0034645; GO:0097659	cellular nitrogen compound biosynthetic process	0.002	BP
GO:0016573; GO:0016570; GO:0018393; GO:0016569; GO:0006475; GO:0018394; GO:0006473; GO:0018205; GO:0043543; GO:0018193; GO:0006325	chromatin organization	0.010	BP
GO:0060026; GO:0003146; GO:0003143; GO:0060562; GO:0070121; GO:0060972; GO:0048793;	convergent extension	0.091	BP

GO:0030433; GO:0036503			
GO:0015074	DNA integration	0.051	BP
GO:0006259	DNA metabolic process	0.078	BP
GO:0006352; GO:0043966; GO:1901796; GO:0042795; GO:0006366; GO:0009301; GO:0098781; GO:0006367; GO:0016032; GO:0044403; GO:0044419	interspecies interaction between organisms	0.000	BP
GO:0031348; GO:0050728	negative regulation of defense response	0.043	BP
GO:2001237	negative regulation of extrinsic apoptotic signaling pathway	0.082	BP
GO:0002832; GO:0050777	negative regulation of immune response	0.088	BP
GO:0050877	nervous system process	0.053	BP
GO:0090501; GO:0090305	nucleic acid phosphodiester bond hydrolysis	0.035	BP
GO:0003002; GO:0007389	pattern specification process	0.051	BP
GO:0043043	peptide biosynthetic process	0.040	BP
GO:0006508	proteolysis	0.053	BP
GO:0070372	regulation of ERK1 and ERK2 cascade	0.075	BP
GO:0040029	regulation of gene expression, epigenetic	0.037	BP
GO:0043523	regulation of neuron apoptotic process	0.043	BP
GO:0032868	response to insulin	0.078	BP
GO:0010243	response to organonitrogen compound	0.078	BP
GO:0009410	response to xenobiotic stimulus	0.080	BP
GO:0016070	RNA metabolic process	0.018	BP
GO:0006396	RNA processing	0.031	BP
GO:0007600	sensory perception	0.088	BP
GO:0007601; GO:0050953	sensory perception of light stimulus	0.012	BP
GO:0002009; GO:0048729; GO:0035239	tissue morphogenesis	0.051	BP

GO:0015629; GO:0000790; GO:0000785; GO:0005669; GO:0090575; GO:0044798; GO:0033276; GO:0070461	RNA polymerase II transcription factor complex	0.000	CC
GO:0043034; GO:0042383	sarcolemma	0.047	CC
GO:0036513; GO:0044322; GO:0000836; GO:0000835; GO:0000153; GO:0000151	ubiquitin ligase complex	0.028	CC
GO:0004190; GO:0070001	aspartic-type endopeptidase activity	0.003	MF
GO:0004190; GO:0070001	aspartic-type peptidase activity	0.061	MF
GO:0034061; GO:0016779; GO:0140097; GO:0003964	catalytic activity, acting on DNA	0.044	MF
GO:0000981; GO:0003700	DNA-binding transcription factor activity	0.074	MF
GO:0004175	endopeptidase activity	0.075	MF
GO:0004519; GO:0004518	nuclease activity	0.071	MF
GO:0070011; GO:0008233	peptidase activity	0.075	MF
GO:0016251; GO:0140223	RNA polymerase II general transcription initiation factor activity	1.998	MF

Supplementary Table 7

Results from pairwise comparisons of Likelihood Ratio tests between larval Kaplan-Meier survivorship fits for each Phenotype~Temperature combination. A=Ambient, H=High, B= Bleached, C= Site-Wide Cross, NB= Nonbleached. p<0.05*; p<0.01**; p<0.001***

	AB	AC	ANB	HB	HC	HNB
Ambient Bleached						
Ambient Cross	***					
Ambient Nonbleached	ns	*				
High Bleached	***	***	***			
High Cross	***	**	***	***		
High Nonbleached	***	***	***	***	***	

Supplementary Table 8

Results from pairwise comparisons of Likelihood Ratio tests between juvenile Kaplan-Meier survivorship fits for each Phenotype~Temperature combination. A=Ambient, H=High, B= Bleached, C= Site-Wide Cross, NB= Nonbleached. $p<0.05$*; $p<0.01$**; $p<0.001$***

	AB	AC	ANB	HB	HC	HNB
Ambient Bleached						
Ambient Cross	***					
Ambient Nonbleached	ns	***				
High Bleached	***	ns	**			
High Cross	***	**	***	***		
High Nonbleached	ns	***	ns	***	***	

Supplementary Table 9
Summary of Gene Ontologies enriched in significantly high differences between nonbleached PBS and cross PBS, isolating functions that are selected significantly more strongly in nonbleached corals and highlighting effects of selective breeding.

Term	Name	P (FDR)	Category
GO:0032230	positive regulation of synaptic transmission, GABAergic	0.030	BP
GO:0032849; GO:0032847	positive regulation of cellular pH reduction	0.030	BP
GO:0004712	protein serine/threonine/tyrosine kinase activity	0.040	MF
GO:0043812; GO:0034596	phosphatidylinositol phosphate 4-phosphatase activity	0.040	MF
GO:0015491; GO:0015298	cation:cation antiporter activity	0.054	MF
GO:0008307	structural constituent of muscle	0.062	MF
GO:0016209; GO:0004601; GO:0016684	antioxidant activity	0.062	MF
GO:0032228	regulation of synaptic transmission, GABAergic	0.078	BP
GO:0051453; GO:0030641; GO:0006885; GO:0030004	regulation of pH	0.078	BP
GO:0008273; GO:0022821	potassium ion antiporter activity	0.083	MF
GO:0032160	septin filament array	0.084	CC
GO:0097733	photoreceptor cell cilium	0.084	CC
GO:0150051	postsynaptic Golgi apparatus	0.084	CC
GO:0006900	vesicle budding from membrane	0.085	BP
GO:0038083	peptidyl-tyrosine autophosphorylation	0.085	BP
GO:0051592	response to calcium ion	0.085	BP
GO:0097553; GO:0060402; GO:0060401	cytosolic calcium ion transport	0.085	BP
GO:0000184	nuclear-transcribed mRNA catabolic process, nonsense-mediated decay	0.099	BP
GO:0007517; GO:0061061	muscle structure development	0.099	BP
GO:0055067	monovalent inorganic cation homeostasis	0.099	BP

Supplementary References

1. Ocampo, I. D., Zárate-Potes, A., Pizarro, V., Rojas, C. A., Vera, N. E., & Cadavid, L. F. (2015). The immunotranscriptome of the Caribbean reef-building coral *Pseudodiploria strigosa*. *Immunogenetics*, *67*(9), 515-530.
2. Matthews, J. L., Crowder, C. M., Oakley, C. A., Lutz, A., Roessner, U., Meyer, E., ... & Davy, S. K. (2017). Optimal nutrient exchange and immune responses operate in partner specificity in the cnidarian-dinoflagellate symbiosis. *Proceedings of the National Academy of Sciences*, *114*(50), 13194-13199.
3. van Oppen, M. J., Bongaerts, P., Frade, P., Peplow, L. M., Boyd, S. E., Nim, H. T., & Bay, L. K. (2018). Adaptation to reef habitats through selection on the coral animal and its associated microbiome. *Molecular ecology*, *27*(14), 2956-2971.
4. Miller, D. J., Hayward, D. C., Reece-Hoyes, J. S., Scholten, I., Catmull, J., Gehring, W. J., ... & Ball, E. E. (2000). Pax gene diversity in the basal cnidarian *Acropora millepora* (Cnidaria, Anthozoa): implications for the evolution of the Pax gene family. *Proceedings of the National Academy of Sciences*, *97*(9), 4475-4480.
5. Smith, E. G., Hume, B. C., Delaney, P., Wiedenmann, J., & Burt, J. A. (2017). Genetic structure of coral-*Symbiodinium* symbioses on the world’s warmest reefs. *PloS one*, *12*(6), e0180169.
6. Aranda, M., Banaszak, A. T., Bayer, T., Luyten, J. R., Medina, M., & Voolstra, C. R. (2011). Differential sensitivity of coral larvae to natural levels of ultraviolet radiation during the onset of larval competence. *Molecular ecology*, *20*(14), 2955-2972.
7. Williams, E. A. (2020). Function and distribution of the Wamide neuropeptide superfamily in metazoans. *Frontiers in endocrinology*, *11*, 344.
8. Heidari, A., Tongsook, C., Najafipour, R., Musante, L., Vasli, N., Garshasbi, M., ... & Vincent, J. B. (2015). Mutations in the histamine N-methyltransferase gene, HNMT, are associated with nonsyndromic autosomal recessive intellectual disability. *Human molecular genetics*, *24*(20), 5697-5710.
9. Yosef, O., Popovits, Y., Malik, A., Ofek-Lalzer, M., Mass, T., & Sher, D. (2020). A tentacle for every occasion: comparing the hunting tentacles and sweeper tentacles, used for territorial competition, in the coral *Galaxea fascicularis*. *BMC genomics*, *21*(1), 1-16.
10. Chiu, Y. L., Shikina, S., Yoshioka, Y., Shinzato, C., & Chang, C. F. (2020). De novo transcriptome assembly from the gonads of a scleractinian coral, *Euphyllia ancora*: molecular mechanisms underlying scleractinian gametogenesis. *BMC genomics*, *21*(1), 1-20.
11. Fuess, L. E., Palacio-Castro, A. M., Butler, C. C., Baker, A. C., & Mydlarz, L. D. (2020). Increased algal symbiont density reduces host immunity in a threatened Caribbean coral species, *Orbicella faveolata*. *Frontiers in Ecology and Evolution*, *8*, 369.
12. Edge, S. E., Shearer, T. L., Morgan, M. B., & Snell, T. W. (2013). Sub-lethal coral stress: detecting molecular responses of coral populations to environmental conditions over space and time. *Aquatic toxicology*, *128*, 135-146.

13. Aguilar, C., Raina, J. B., Fôret, S., Hayward, D. C., Lapeyre, B., Bourne, D. G., & Miller, D. J. (2019). Transcriptomic analysis reveals protein homeostasis breakdown in the coral *Acropora millepora* during hypo-saline stress. *BMC genomics*, *20*(1), 1-13.
14. Alves, C. J., Yotoko, K., Zou, H., & Friedel, R. H. (2019). Origin and evolution of plexins, semaphorins, and Met receptor tyrosine kinases. *Scientific reports*, *9*(1), 1-14.
15. Dunn, S. R., Schnitzler, C. E., & Weis, V. M. (2007). Apoptosis and autophagy as mechanisms of dinoflagellate symbiont release during cnidarian bleaching: every which way you lose. *Proceedings of the Royal Society B: Biological Sciences*, *274*(1629), 3079-3085.
16. Cheng, J., Moyer, B. D., Milewski, M., Loffing, J., Ikeda, M., Mickle, J. E., ... & Guggino, W. B. (2002). A Golgi-associated PDZ domain protein modulates cystic fibrosis transmembrane regulator plasma membrane expression. *Journal of Biological Chemistry*, *277*(5), 3520-3529.
17. Hemond, E. M., Kaluziak, S. T., & Vollmer, S. V. (2014). The genetics of colony form and function in Caribbean *Acropora* corals. *BMC genomics*, *15*(1), 1-21.
18. Takeuchi, T., Yamada, L., Shinzato, C., Sawada, H., & Satoh, N. (2016). Stepwise evolution of coral biomineralization revealed with genome-wide proteomics and transcriptomics. *PloS one*, *11*(6), e0156424.
19. DeSalvo, M. K., Voolstra, C. R., Sunagawa, S., Schwarz, J. A., Stillman, J. H., Coffroth, M. A., ... & Medina, M. (2008). Differential gene expression during thermal stress and bleaching in the Caribbean coral *Montastraea faveolata*. *Molecular ecology*, *17*(17), 3952-3971.
20. Mayfield, A. B., Chen, Y. J., Lu, C. Y., & Chen, C. S. (2018). The proteomic response of the reef coral *Pocillopora acuta* to experimentally elevated temperatures. *PloS one*, *13*(1), e0192001.
21. DeSalvo, M. K., Sunagawa, S., Voolstra, C. R., & Medina, M. (2010). Transcriptomic responses to heat stress and bleaching in the elkhorn coral *Acropora palmata*. *Marine Ecology Progress Series*, *402*, 97-113.
22. Alzugaray, M. E., Hernández-Martínez, S., & Ronderos, J. R. (2016). Somatostatin signaling system as an ancestral mechanism: Myoregulatory activity of an Allatostatin-C peptide in Hydra. *Peptides*, *82*, 67-75.
23. Mansfield, K. M., Cleves, P. A., Van Vlack, E., Kriefall, N. G., Benson, B. E., Camacho, D. J., ... & Gilmore, T. D. (2019). Varied effects of algal symbionts on transcription factor NF-κB in a sea anemone and a coral: possible roles in symbiosis and thermotolerance. *bioRxiv*, 640177.
24. Levy, O., Karako-Lampert, S., Ben-Asher, H. W., Zoccola, D., Pagès, G., & Ferrier-Pagès, C. (2016). Molecular assessment of the effect of light and heterotrophy in the scleractinian coral *Stylophora pistillata*. *Proceedings of the Royal Society B: Biological Sciences*, *283*(1829), 20153025.

25. Panevska, A., Skočaj, M., Križaj, I., Maček, P., & Sepčić, K. (2019). Ceramide phosphoethanolamine, an enigmatic cellular membrane sphingolipid. *Biochimica et Biophysica Acta (BBA)-Biomembranes*, *1861*(7), 1284-1292.
26. Doonan, L. B., Hartigan, A., Okamura, B., & Long, P. F. (2019). Stress-free evolution: the Nrf-coordinated oxidative stress response in early diverging metazoans. *Integrative and comparative biology*, *59*(4), 799-810.
27. Cunning, R., Bay, R. A., Gillette, P., Baker, A. C., & Traylor-Knowles, N. (2018). Comparative analysis of the *Pocillopora damicornis* genome highlights role of immune system in coral evolution. *Scientific Reports*, *8*(1), 1-10.
28. Kwong, W. K., del Campo, J., Mathur, V., Vermeij, M. J., & Keeling, P. J. (2019). A widespread coral-infecting apicomplexan with chlorophyll biosynthesis genes. *Nature*, *568*(7750), 103-107.
29. Komander, D. (2009). The emerging complexity of protein ubiquitination. *Biochemical Society Transactions*, *37*(5), 937-953.
30. Liebl, F. L., Werner, K. M., Sheng, Q., Karr, J. E., McCabe, B. D., & Featherstone, D. E. (2006). Genome-wide P-element screen for *Drosophila synaptogenesis* mutants. *Journal of neurobiology*, *66*(4), 332-347.
31. Shahnazari, S., & Brumell, J. H. (2009). Eating twice for the sake of immunity: a phagocytic receptor that activates autophagy. *Cell host & microbe*, *6*(4), 297-298.
32. Li, M., Liu, H., Guo, Y., Chen, F., Zi, X., Fan, R., ... & Zhao, X. (2020). Single symbiotic cell transcriptome sequencing of coral. *Genomics*, *112*(6), 5305-5312.
