# Supplementary material for: Intrapopulation adaptive variance supports thermal tolerance in a reef-building coral
Source: Commun Biol. 2022 May 19;5:486. doi: 10.1038/s42003-022-03428-3 (PMC9120509; doi:10.1038/s42003-022-03428-3)
Supplement: Supplementary file 2 — Reporting Summary [file 42003_2022_3428_MOESM2_ESM.pdf]

nature portfolio

Corresponding author(s): Crawford DRury

Last updated by author(s): Apr 15, 2022

Reporting Summary

Nature Portfolio wishes to improve the reproducibility of the work that we publish. This form provides structure for consistency and transparency in reporting. For further information on Nature Portfolio policies, see our Editorial Policies and the Editorial Policy Checklist.

Statistics

For all statistical analyses, confirm that the following items are present in the figure legend, table legend, main text, or Methods section.

n/a	Confirmed	
☐	☒	The exact sample size (*n*) for each experimental group/condition, given as a discrete number and unit of measurement
☐	☒	A statement on whether measurements were taken from distinct samples or whether the same sample was measured repeatedly
☐	☒	The statistical test(s) used AND whether they are one- or two-sided<br>*Only common tests should be described solely by name; describe more complex techniques in the Methods section.*
☒	☐	A description of all covariates tested
☐	☒	A description of any assumptions or corrections, such as tests of normality and adjustment for multiple comparisons
☐	☒	A full description of the statistical parameters including central tendency (e.g. means) or other basic estimates (e.g. regression coefficient) AND variation (e.g. standard deviation) or associated estimates of uncertainty (e.g. confidence intervals)
☐	☒	For null hypothesis testing, the test statistic (e.g. *F*, *t*, *r*) with confidence intervals, effect sizes, degrees of freedom and *P* value noted<br>*Give P values as exact values whenever suitable.*
☒	☐	For Bayesian analysis, information on the choice of priors and Markov chain Monte Carlo settings
☒	☐	For hierarchical and complex designs, identification of the appropriate level for tests and full reporting of outcomes
☒	☐	Estimates of effect sizes (e.g. Cohen's *d*, Pearson's *r*), indicating how they were calculated

Our web collection on statistics for biologists contains articles on many of the points above.

Software and code

Policy information about availability of computer code

Data collection	Larval size was measured in Adobe Photoshop CC 2018. Microscope photos were taken with an Amscope MU3000 using Amscope 3.7.6701.
Data analysis	The following software was used to align, call SNPs and allele frequencies and process gene ontology data: Bowtie2 2.3.5.1, ANGSD 0.929, GATK 4.1.2.0, BLAST2GO 5.2.5<br>The following software and packages were used to analyze data:<br>JMP Pro 14, R 3.5, Rstudio 1.1.453, R packages: Tidyverse, readxl, survival, surmviner, cowplot, plotrix, car, zoo, adegenet, R.utils, vcfR, naniar, lsmeans, ade4, hierfstat, GO_MWU (https://github.com/z0on/GO_MWU)

For manuscripts utilizing custom algorithms or software that are central to the research but not yet described in published literature, software must be made available to editors and reviewers. We strongly encourage code deposition in a community repository (e.g. GitHub). See the Nature Portfolio guidelines for submitting code & software for further information.

Data

Policy information about availability of data

All manuscripts must include a data availability statement. This statement should provide the following information, where applicable:

- Accession codes, unique identifiers, or web links for publicly available datasets
- A description of any restrictions on data availability
- For clinical datasets or third party data, please ensure that the statement adheres to our policy

All analysis scripts, ecological data and processed sequencing data are available at github.com/druryc/mcap_bleaching. Raw sequencing data is available at NCBI

PRJNA597077. Feature-based molecular networking is available at: https://gnps.ucsd.edu/ProteoSAFe/status.jsp?task=bb9b6126118c4ba1881f4483560012d3 and raw files are available at massive.ucsd.edu under MassIVE ID MSV000085272 and MSV000085925.

Field-specific reporting

Please select the one below that is the best fit for your research. If you are not sure, read the appropriate sections before making your selection.

☐ Life sciences ☐ Behavioural & social sciences ☒ Ecological, evolutionary & environmental sciences

For a reference copy of the document with all sections, see nature.com/documents/nr-reporting-summary-flat.pdf

Ecological, evolutionary & environmental sciences study design

All studies must disclose on these points even when the disclosure is negative.

Study description	This study uses adults and gametes collected from 22 coral colonies, pooled by historical bleaching phenotype (n=11 colonies per phenotype) and combined to form a control cross. These materials were treated at two temperatures (ambient and high), so that phenotypic pool and temperature were treated as fully crossed factors in statistical analysis. Experimental units for treatments were 150L conicals, experimental units for larval survivorship were 50mL conical tubes with 50 larvae (n=7-15 replicates), experimental units for allele frequency over time was pooled samples of 50 larvae (n=5 replicates), experimental units for adult SNPs was a coral colony (n=11 per phenotype), experimental unit for juvenile treatment was plug (n=17-43 per phenotype).
Research sample	This study sampled 22 adult Montipora capitata and their gametes from a single reef in K?ne?ohe Bay, Hawaii. This population is meant to represent a major reef-building coral in this ecosystem at a naturally realistic spatial scale that is relevant for sexual reproduction and recruitment.
Sampling strategy	Sampling was limited by logistical constraints. We attempted to maximize the number of source colonies that were accessible in situ during night-time spawning. Replication for larval survivorship (n=15) was arbitrary, replication for juvenile survivorship was based on maximum available number of settlers.
Data collection	Gamete and adult samples were collected by the authors (CD, JH, NB, CH). Survivorship data was collected by NB, CH, JH. Symbiont samples were collected and processed by CH. Sequencing samples were collected by CD. Sequencing processing and data analysis was completed by CD.
Timing and spatial scale	Data on adult colony bleaching that informed the study design was collected in 2015 based on a natural bleaching event. The timing of this experiment was dictated by natural coral spawning events, with samples collected on a single night, 13 July 2018. Larval survivorship estimates were conducted as frequently as possible for the first phase of the experiment, daily. Juvenile survivorship estimates were conducted weekly to efficiently capture declines. The spatial scale of adult colonies spans approximately 120 linear meters, deliberately chosen to represent coral colonies that would naturally reproduce on a single reef.
Data exclusions	Loci were excluded from the study if less than 3 replicates generated allele frequency results. This allows low-coverage loci to be retained in the study, but only if replication was available, strengthening the confidence in frequency calls. Standard, but arbitrary cutoffs for missingness were used in sequencing analysis (e.g. <10% missingness for clonality analysis, <10% change in allele frequency in ambient conditions to highlight selection, etc.)
Reproducibility	Due to the logistical difficulty of field collections, no efforts were made to reproduce the experiment.
Randomization	Adult samples were collected from haphazardly within colony. Larvae were pooled across phenotype.
Blinding	Blinding was not relevant to our study, data collected were based on raw counts and haphazard sampling.

Did the study involve field work? ☒ Yes ☐ No

Field work, collection and transport

Field conditions	Field gamete collections were made at a single shallow reef on 13 July 2018. Adult branches were sampled for sequencing in late July 2018 and for metabolites in May 2019.
Location	Study materials were collected from Reef 13 in K?ne?ohe Bay within 50m of 21.4511356, -157.796417. All colonies in the experiment were at 2-4m depth
Access & import/export	The site was accessed by boat and did not interfere with the environment. Adult and gamete collections were made under Hawaii Department of Land and Natural Resources permit SAP 2018-03 issued to the Hawaii Institute of Marine Biology on 1/23/2017.
Disturbance	The use of weighted conical mesh nets for gamete collections minimized impact on adult colonies by avoiding sharp or hard surfaces. Haphazard branch collection for symbiont and genomics from the parents followed standard, permit approved protocols to limit sampling damage, collecting a minimum amount of material needed.

Reporting for specific materials, systems and methods

We require information from authors about some types of materials, experimental systems and methods used in many studies. Here, indicate whether each material, system or method listed is relevant to your study. If you are not sure if a list item applies to your research, read the appropriate section before selecting a response.

Materials & experimental systems

n/a	Involved in the study
☒	☐ Antibodies
☒	☐ Eukaryotic cell lines
☒	☐ Palaeontology and archaeology
☐	☒ Animals and other organisms
☒	☐ Human research participants
☒	☐ Clinical data
☒	☐ Dual use research of concern

Methods

n/a	Involved in the study
☒	☐ ChIP-seq
☒	☐ Flow cytometry
☒	☐ MRI-based neuroimaging

Animals and other organisms

Policy information about studies involving animals; ARRIVE guidelines recommended for reporting animal research

Laboratory animals	This study did not involve laboratory animals
Wild animals	22 adult Montipora capitata coral colonies (hermpahordites) were netted to collect gametes. Adult colonies were not removed and sampling was non-invasive.
Field-collected samples	Gametes were collected and fertilized in ambient seawater (~27C). After fertilization, 12 hours at ambient temperatures was follwed by temperature treatments (high ~30.5C and ambient ~28C). The experiment was conducted under natural sunlight with 70% shade cloth.
Ethics oversight	Aside from colleciton permits, no ethical oversight is required for working with invertebrates in the state of Hawai..

Note that full information on the approval of the study protocol must also be provided in the manuscript.
